# Iron Deficiency Reprograms Phosphorylation Signaling and Reduces O-GlcNAc Pathways in Neuronal Cells

**DOI:** 10.3390/nu13010179

**Published:** 2021-01-08

**Authors:** Luke N. Erber, Ang Luo, Yao Gong, Montana Beeson, Maolin Tu, Phu Tran, Yue Chen

**Affiliations:** 1 Department of Biochemistry, Molecular Biology and Biophysics, University of Minnesota at Twin Cities, Minneapolis, MN 55455, USA; lerber@umn.edu (L.N.E.); luoang369@nwafu.edu.cn (A.L.); gong0062@umn.edu (Y.G.); tumaolin012@163.com (M.T.); 2 Department of Pediatrics, University of Minnesota at Twin Cities, Minneapolis, MN 55455, USA; beeso025@umn.edu (M.B.); tranx271@umn.edu (P.T.)

**Keywords:** oxygen sensing, hypoxia, iron deficiency, quantitative proteomics, phosphorylation, neuronal cells, HT22, hippocampal cells

## Abstract

Micronutrient sensing is critical for cellular growth and differentiation. Deficiencies in essential nutrients such as iron strongly affect neuronal cell development and may lead to defects in neuronal function that cannot be remedied by subsequent iron supplementation. To understand the adaptive intracellular responses to iron deficiency in neuronal cells, we developed and utilized a Stable Isotopic Labeling of Amino acids in Cell culture (SILAC)-based quantitative phosphoproteomics workflow. Our integrated approach was designed to comprehensively elucidate the changes in phosphorylation signaling under both acute and chronic iron-deficient cell models. In addition, we analyzed the differential cellular responses between iron deficiency and hypoxia (oxygen-deprived) in neuronal cells. Our analysis identified nearly 16,000 phosphorylation sites in HT-22 cells, a hippocampal-derived neuronal cell line, more than ten percent of which showed at least ≥2-fold changes in response to either hypoxia or acute/chronic iron deficiency. Bioinformatic analysis revealed that iron deficiency altered key metabolic and epigenetic pathways including the phosphorylation of proteins involved in iron sequestration, glutamate metabolism, and histone methylation. In particular, iron deficiency increased glutamine-fructose-6-phosphate transaminase (GFPT1) phosphorylation, which is a key enzyme in the glucosamine biosynthesis pathway and a target of 5′ AMP-activated protein kinase (AMPK), leading to reduced GFPT1 enzymatic activity and consequently lower global O-GlcNAc modification in neuronal cells. Taken together, our analysis of the phosphoproteome dynamics in response to iron and oxygen deprivation demonstrated an adaptive cellular response by mounting post-translational modifications that are critical for intracellular signaling and epigenetic programming in neuronal cells.

## 1. Introduction

Iron deficiency (ID) is one of the most prevalent micronutrient deficiencies, affecting approximately 30% of pregnant women and pre-school age children worldwide, and it causes poor long-term neurodevelopment outcomes and increased risks of psychiatric disorders in later life [1,2]. Iron is an essential nutrient for cell development and function [3]. Chronic ID leads to anemia and affects brain development, particularly during the rapid growth period that spans the late third trimester of fetal life and early childhood. Early-life (fetal and early postnatal) ID anemia causes neurodevelopment deficits, including in learning and memory, which are not fully rescued by the subsequent iron supplementation and resolution of ID anemia [4,5,6]. Brain regions related to cognitive development such as the hippocampus are strongly impacted by ID [7,8,9,10,11]. Collectively, insufficient iron uptake from the cellular microenvironment can lead to ID and thereby abnormal growth and development [12,13]. Thus, elucidating the iron-sensitive targets that regulate intracellular signaling constitutes an important step toward the development of more effective therapeutic approaches.

The ability of cells to detect nutrient deficiencies such as iron constitutes an early cellular response and plays a critical role in maintaining normal physiology and developmental activity [13,14,15]. Such an early response involves complex and diverse signaling mechanisms that serve as cellular “sensors” to the extracellular cues [15,16,17]. For instance, a loss of iron uptake alters neuronal mitochondria activity and induces global changes of gene expression through cell signaling and epigenetic mechanisms including Brain-derived neurotrophic factor (BDNF), Hypoxia-inducible factor 1-alpha (HIF1a), and mammalian target of rapamycin (mTOR) pathways [18,19,20]. To date, system-wide analysis of ID in mammalian cells and tissues has been largely limited to the overall changes in the transcriptome and proteome [21,22,23,24]. These studies revealed dynamic changes in gene expression and protein levels related to cellular energy metabolism, growth arrest, DNA damage, neuronal growth, and epigenetic modifications. Despite these advances, there is a lack of system-wide understanding of how phosphorylation-dependent cellular signaling is affected by ID in neuronal cells.

In the present study, we applied a system-wide phosphoproteomic screening in combination with Stable Isotopic Labeling of Amino acids in Cell culture (SILAC) [25] to identify changes in phosphorylation signaling pathways in response to both acute and chronic ID in hippocampal neuronal cells. In addition, there is a strong molecular link between ID and hypoxia. Iron is a key co-factor for numerous oxygen-dependent enzymes and cellular processes. ID significantly reduces the enzymatic activity of di-oxygenases including prolyl hydroxylases (PHDs) that hydroxylate hypoxia-inducible factor 1 alpha (HIF1a), which is a major transcription factor that regulates the cellular hypoxia response pathway [26,27,28,29,30]. Both ID and hypoxia reduce HIF1a hydroxylation and degradation, thereby resulting in the upregulation of HIF1a-dependent gene expression [14]. To further distinguish the effects of ID from hypoxia in cellular signaling, we performed quantitative phosphoproteomics analysis of hypoxia-response phosphoproteome in neuronal cells. Our integrated workflow provided new insights on the ID- and hypoxia-induced dynamic changes of metabolic pathways and epigenetic programming. We identified diverse signaling mechanisms that were differentially regulated by ID and hypoxia in hippocampal neuronal cells.

## 2. Materials and Methods

### 2.1. Animals

All experiments were approved by the University of Minnesota Institutional Animal Care and Use Committee. Brain ID was induced in rat pups using an iron-deficient diet regimen described previously [31]. In brief, timed-pregnant Sprague–Dawley dams (Charles River, Wilmington, MA, USA) were randomly assigned to either iron-deficient (2–6 ppm iron, TD.80396, Envigo, Indiana, IN, USA) or iron-sufficient (200 ppm iron, TD.09256, Envigo, Indianapolis, IN, USA) fortified diets ad libitum from gestational day 3 until postnatal day 7 (P7). Shortly after birth, litters were culled to 8 pups containing at least 2 females. On P7, all dams were placed on the iron-sufficient diet. On P15, pups were sacrificed, and hippocampi were micro-dissected, flash-frozen in liquid nitrogen, and stored at −80 °C until use.

### 2.2. Cell Culture and SILAC Labeling

HT22 cells (A gift from Dr. Schubert, Salk Institute, La Jolla, CA, USA) were cultured in Dulbecco’s Modified Eagle Medium (DMEM) (Gibco, Waltham, MA, USA) supplemented with 10% fetal bovine serum (Sigma, St. Louis, MO, USA) and 1% penicillin–streptomycin (Corning, Glendale, AZ, USA). For SILAC labeling, the HT22 cells were maintained in DMEM for SILAC (Thermo, Waltham, MA, USA), which was supplemented with 10% dialyzed fetal bovine serum (FBS) (Gibco), 1% penicillin–streptomycin, 25 mg/500 mL proline, and 50 mg/500 mL l-arginine and l-lysine (light) or ^13^C_6_^15^N_4_-l-arginine and ^13^C_6_^15^N_2_-l-lysine (heavy). The cells were labeled respectively in the light and heavy media for more than 6 generations before additional treatment. Cells were maintained in a humidified incubator at 3 °C and 5% CO_2_. For cell treatments, deferoxamine (Sigma-Aldrich, St. Louis, MO, USA) was solubilized in DMSO. Upon treatment, the final concentration of DMSO was 0.7%. For acute iron deficiency treatment, HT22 cells were treated with 100 µM deferoxamine for 6 h. For chronic iron deficiency treatment, HT22 cells were treated with 10 µM deferoxamine for 24 h. For the hypoxia treatment, HT22 cells were incubated at 37 °C for 6 h in a hypoxia chamber (1% O_2_, 94% N_2_, 5% CO_2_). For the normoxia treatment, HT22 cells were incubated at 37 °C for 6 h in a humidified incubator (18% O_2_, 77% N_2_, 5% CO_2_).

### 2.3. Cell and Tissue Lysates

For phosphoproteomic analysis, SILAC-labeled HT22 cells were harvested at approximately 80% confluency by washing twice with cold phosphate-buffered saline (Gibco, Waltham, MA, USA) and subsequently adding boiling lysis buffer (6 M guanidine hydrochloride (GuHCl), 100 mM Tris, pH 8.5, protease inhibitor, phosphatase inhibitor) directly to the plate. Cells were collected by scraping the plate and immediately boiled for an additional 10 min followed by micro tip sonication. The lysate was subjected to high-speed centrifugation for 10 min.

For WB analysis, HT22 cells were lysed in ice-cold RIPA buffer (150 mM NaCl, 50 mM Tris–HCl, pH 7.5, 0.1% sodium dodecyl sulfate (SDS), 0.5% NP-40, and 0.5% sodium deoxycholate) with freshly prepared complete cocktail protease inhibitor (Roche, Basel, Switzerland).

Flash-frozen hippocampus was lysed in 300 µl cytoskeletal protein lysis buffer (10 mM Tris pH 7.4, 100 mM NaCl, 1 mM ethylenediaminetetraacetic acid (EDTA), 1 mM ethylene glycol tetraacetic acid (EGTA), 1 mM NaF, 20 mM Na_4_P_2_O_7_, 2 mM Na_3_VO_4_, 1% Triton x-100, 10% glycerol, 0.1% SDS, 0.5% deoxycholate) with complete protease inhibitor cocktails (Roche) using a Kimble pellet pestle motor (Grainger Industry, Lake Forest, IL, USA). Then, lysates were sonicated using a Bioruptor Pico (Diagenode, Denville, NJ, USA), centrifuged at 10,000 rpm 5 min, and stored at −80 °C until use.

### 2.4. Western Blotting Analysis

Protein concentration of the cell lysate was determined using Bradford assay (Thermo). Proteins were suspended in SDS loading buffer and boiled for 8 min. Then, 30 µg of proteins were separated in SDS-PAGE gel and transferred onto polyvinylidene difluoride (PVDF) membrane. Blotted membrane was blocked in 5% non-fat skim milk (BD) diluted in TBST (Tris-buffered saline +0.1% Tween 20). After blocking, the membrane was incubated with primary antibody overnight and washed with TBST at least three times and then incubated with horseradish peroxidase (HRP)-linked secondary antibody (Cell Signaling Technology, #7074 and #7076, Danvers, MA, USA) for >2 h and washed with TBST. The signal was developed with Luminata Crescendo Western HRP Substrate (Millipore, Burlington, MA, USA) and captured on X-ray film. Primary antibodies in this study include the Anti-O-Linked N-Acetylglucosamine antibody [RL2] (ab2739, Abcam, Cambridge, UK), anti-Tubulin (T6199, Sigma), and anti-HIF1a (04-1006, Millipore, Burlington, MA, USA). Blot densitometry quantified with ImageJ. O-GlcNAc and HIF1a signal was normalized against tubulin.

### 2.5. Quantitative PCR Analysis

To induce cellular iron deficiency, 700,000 HT22 cells were seeded onto a 60 mm plate (Costar, Washington, DC, USA). At 70% confluency, cells were incubated in DMEM growth media with 10 or 100 µM iron chelator deferoxamine (DFO) for 8, 24, and 96 h. The cellular iron deficiency was assessed by the upregulation of transferrin receptor (TfR1) using Taqman gene expression assay (Mm00441941_m1, ABI, Carlsbad, CA, USA) for Real-time PCR. In brief, following a PBS rinse, RNA was isolated from adherent cells with 0.5 mL RNA lysis buffer (RNAqueous Phenol-free total RNA Isolation Kit, Invitrogen) and purified following manufacturer’s protocol (Invitrogen). Then, 1.0 µg of total RNA was used for cDNA synthesis (High Capacity RNA-to-cDNA Kit, ABI). Then, cDNA was diluted 10× and quantified for TfR1 levels using a QuantStudio 3 Real-Time PCR system (ThermoFisher, Waltham, MA, USA). Samples (n = 3/treatment) were analyzed in duplicates. TATA-box binding protein (Tbp, Mm01277042_m1) or rps18 (Mm02601777_g1) was used as an internal control. The expression of either internal control gene was not changed by DFO treatment.

### 2.6. Peptide Preparation

Protein concentration was estimated by Bradford assay (Thermo, Waltham, MA, USA). The heavy and light SILAC-labeled lysates were mixed in a 1:1 *w*/*w* ratio. Proteins were reduced and alkylated with tris(2-carboxyethyl)phosphine (TCEP) (5 mM) and iodoacetamide (5 mM) followed by blocking with cysteine (5 mM). Protein sample was diluted to 1 M GuHCl using 50 mM Tris, pH 8.5. The sample was adjusted to pH 8.0 with 5 mM ammonium bicarbonate for tryptic digestion. Protein was digested with trypsin (Promega, Madison, WI, USA) in an enzyme/protein ratio of 1:50 (*w*/*w*) overnight at 37 °C and further digested with trypsin for 2 h at a ratio of 1:100 (*w*/*w*) at 37 °C. The sample was centrifuged at 2000 rpm for 10 min, and the resulting peptide mixture was concentrated using reversed-phase Sep-Pak C18 Cartridge (Waters, Milford, MA, USA). Peptides were eluted off the Sep-Pak with 1.2 mL 80% acetonitrile (ACN). The ACN was removed by vacuum centrifugation. Then, the peptides were stored at −80 °C.

### 2.7. Offline High pH Reversed-Phase HPLC Fractionation

First, 4–5 mg of peptides were resuspended in 10 mM ammonia formate (pH 8.0) and fractionated using a Waters XBridge peptide BEH C18 column (3.5 μm, 4.6 mm × 150 mm) on an Agilent 1100 HPLC system (Agilent, Santa Clara, CA, USA) operating at a flow rate of 1 mL/min with 2 buffer lines: buffer A (10 mM ammonia formate in water, pH 10.0) and buffer B (10 mM ammonia formate, pH 10.0 and 90% ACN). Peptides were separated by a linear gradient from 3% B to 35% B for 45 min followed by a linear increase to 95% B for 8 min and decreased to 3% B for 2 min and maintained for 5 min. Fractions were collected at 60 s intervals for a total of 4 concatenated fractions. Samples were lyophilized and desalted using Thermo Pierce peptide desalting spin columns. Ten percent of the peptide samples were saved for quantitative proteome analysis, and the remaining was used for phosphopeptide enrichment.

### 2.8. Phosphopeptide Enrichment

Phosphopeptides from 1 mg of each peptide fraction were enriched using the High-Select Fe-NTA phosphopeptide enrichment kit from Thermo Fisher Scientific (Waltham, MA, USA). Eluted peptides were dehydrated using a speed-vac and desalted using homemade C18 Stage-tips.

### 2.9. LC-MS/MS Acquisition

Mass spectrometry (MS) experiments were performed on an Orbitrap Fusion mass spectrometer (Thermo Scientific) connected to an online Proxeon Easy nLC 1000 Nano-UPLC system (Thermo Scientific). Peptides were resolubilized in HPLC buffer A (0.1% formic acid in water, *v*/*v*) and loaded onto a self-packed capillary HPLC column (50 cm × 100 µm, ReproSil-Pur Basic C18, 2.5 µm, Dr. Maisch GmbH) heated at 55 °C. Peptides were separated by the Proxeon nLC system at a flow rate of 300 nL/min with a gradient consisting of 79 min of 5–22% HPLC buffer B (0.1% formic acid in acetonitrile, *v*/*v*), 11 min of 22–32% buffer B, and 10 min of 32–95% buffer B.

Precursor ions were ionized using electrospray and detected by the orbitrap at a resolution of 120,000 at 200 *m*/*z* and a mass range of 380–1800 *m*/*z*. The precursor ions were filtered using a dynamic exclusion duration of 15 s and a mass tolerance of ± 25 ppm. Following fragmentation using high energy collisional dissociation (HCD) of 30%, fragment ions were acquired in the linear ion trap with an isolation window of 1.6 *m*/*z*.

### 2.10. Sequence Database Searching and Data Processing

Mass spectrometry data were analyzed with MaxQuant software (version 1.5.3.12). Peptides were identified using the integrated Andromeda search engine with default settings against the UniProt database for mouse at a 1% false discovery rate (FDR). Carbamidomethylation of cysteine residues was set as fixed, whereas the acetylation of protein N-termini, oxidation of methionine, and phosphorylation of serine, threonine, and tyrosine were specified as variable modifications. For SILAC quantification, the multiplicity was set at two with heavy labeled Arg10 and Lys8 selected.

### 2.11. Functional Annotation and Clustering

To carry out clustering analysis, we divided the data into four quantiles according to their normalized SILAC Heavy/Light (H/L) ratio. The quantiles were divided into four log_2_ ratio ranges: less than −1, −1 to 0, 0 to 1, and greater than 1, respectively. We performed statistical enrichment analysis for each quantile using a hypergeometric test with the following R packages: GO.db, GOstats, and org.Mm.eg.db. We performed enrichment analysis for the Kyoto Encyclopedia of Genes and Genomes (KEGG) pathway, Pfam domains, and Gene Ontology—biological process, molecular function, and cellular compartment. We calculated the −log_10_ of the *p*-value outcome of the enrichment and normalized them to calculate the z-score. The *p*-value cut off for significance is 0.05, and the z-score cut off for significance is 1.2. For each category, we used one-way hierarchical clustering (average linking and covariance value as distance) of the annotation based on the z-score in Genesis software.

### 2.12. Kinase Activity Profiling Analysis (KAPA)

To perform Kinase Activity Profiling Analysis, we first downloaded the kinase-substrate annotation database from PhosphoSitePlus (www.phosphosite.org) [32]. Then, we normalized the phosphorylation SILAC H/L ratios in our dataset with their corresponding protein SILAC H/L ratios. Only quantifiable phosphorylation sites after normalization were kept for the Kinase Activity Profiling Analysis (KAPA) study. To assess the activity of each kinase, we extracted the site-specific phosphorylation SILAC H/L ratios of identified kinase substrates based on the kinase–substrate annotation profile. These ratios were log_2_ transformed, and the average log_2_ ratio of the kinase targets was calculated. Next, we performed bootstrapping-based random sampling. For *k* target sites of a specific kinase quantified in this study, we randomly selected *k* quantifiable phosphorylation sites from our phosphorylation dataset, and the average log_2_ ratio of the randomly selected phosphorylation sites was calculated. Then, the random selection process was repeated 1000 times. The average log_2_ ratios from these random selections will form a normal distribution based on the Central Limit Theorem. Finally, from this normal distribution, we calculated the z-score for the average log_2_ ratio of the specific kinase target sites, and the z-score was termed as the Kinase Activity Score described below. An in-house developed Perl script was applied to perform Kinase Activity Profiling Analysis.

Kinase Activity Score = (*S* − *Ave*)/*Std*

*S* stands for the average log_2_ ratio of normalized phosphorylation target sites of a specific kinase, *Ave* and *Std* stand for the average and standard deviation of the normal distribution formed by the average log_2_ SILAC H/L ratios of 1000 random samplings of *k* phosphorylation sites from the phosphorylation dataset, while *k* equals the number of target sites for a specific kinase quantified in the study.

## 3. Results

### 3.1. Experimental Strategy for the Quantification of the Iron- and Oxygen-Starvation Dependent Proteome in Neuronal Cells

**Figure 1:**
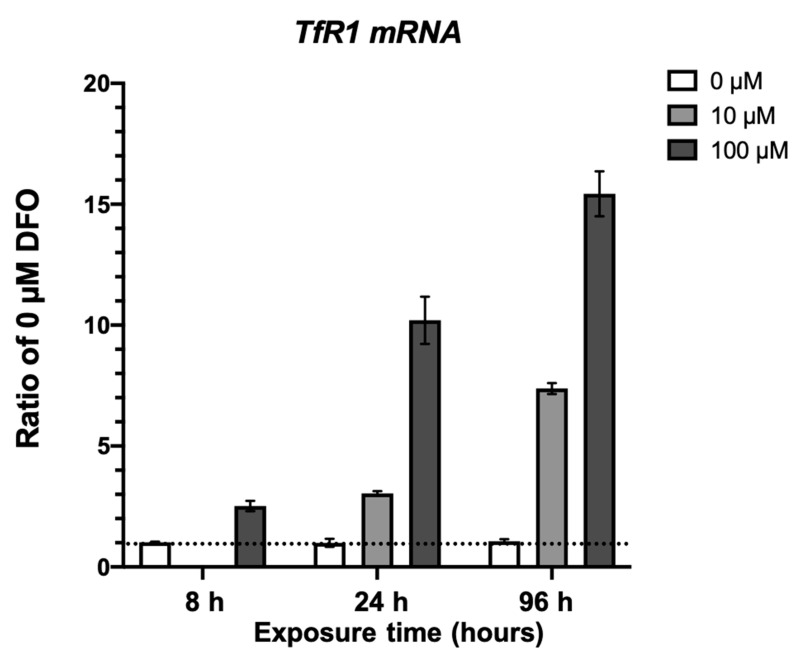
Quantitative PCR analysis of *TfR1* expression. HT22 cells were treated with 10 or 100 µM iron chelator deferoxamine (DFO) for 8, 24, and 96 h. The cellular iron deficiency was assessed by the upregulation of transferrin receptor 1 using quantitative real-time PCR. Samples (n = 3/treatment) were analyzed in duplicates and TATA-box binding protein (Tbp) or rps18 were used for normalization. Mixed-effects model 2-way ANOVA, *p* = 0.0001, F(2,16) = 17.12.

To study the dynamics of cellular signaling pathways sensitive to oxygen and iron starvation in neurons, we analyzed phosphoproteomic changes in HT22 cells, an established mouse hippocampal cell line [33], in response to hypoxia and deferoxamine (DFO) treatments. Deferoxamine is a well-characterized cell-permeable iron chelator that has been widely used to treat the cells and induce iron deficiency. To demonstrate that deferoxamine treatment effectively chelates Fe to induce iron deficiency in our system, HT22 cells were treated with 0 µM, 10 µM, and 100 µM at increasing timepoints. We used the expression of transferrin receptor 1 (*TfR1*) as a marker indexing cellular iron deficiency [8]. Quantitative PCR analysis revealed the *TfR1* mRNA levels increase in response DFO treatment (Figure 1). Furthermore, *TfR1* mRNA levels respond differentially to 0, 10, and 100 µM treatment at 8, 24, and 96 h.

**Figure 2:**
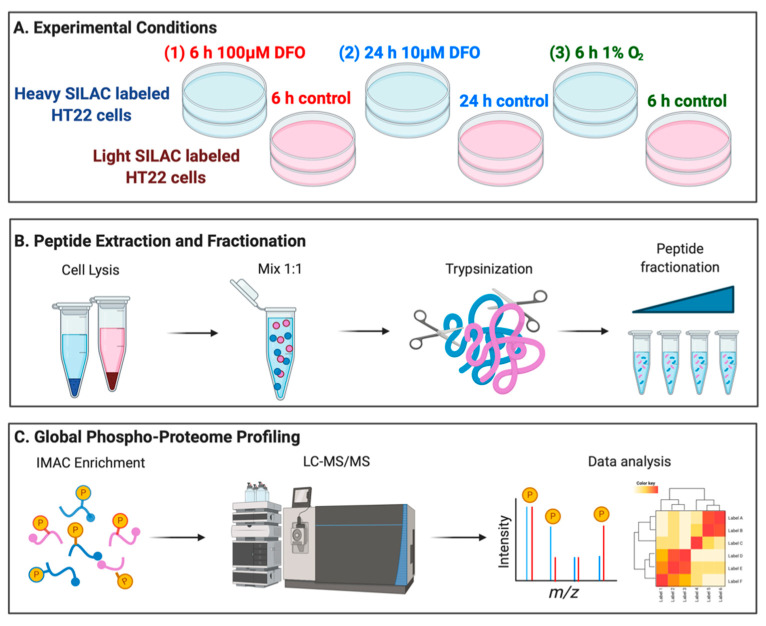
Experimental design and phosphoproteomic workflow for comprehensive analysis of phosphorylation proteome. (**A**) Outline of experimental workflow using Stable Isotopic Labeling of Amino acids in Cell culture (SILAC)-labeled HT22 cells, (**B**) generating peptides for quantification, and (**C**) enrichment of phospho-sites, mass spectrometry acquisition, and data analysis.

In experimental studies, cellular iron deficiency could be achieved with either a high dose of DFO treatment for a short period of time (4–8 h, 100 µM to 1 mM) [34,35,36], or a low dose of DFO treatment for a relatively long period of time (24–48 h, 10–20 µM) [3,37,38,39]. We consider the former condition as acute iron deficiency and the latter condition as chronic iron deficiency, and the differential effects of either treatment remain largely unknown. To systematically reveal ID-induced early phosphorylation dynamics, we included both acute and chronic ID in our study, which also models physiological conditions based on the extent of transferrin receptor upregulation. In addition, we performed a similar analysis of cells with acute hypoxia treatment to isolate the differential outcomes of oxygen starvation versus ID in HT22 cells (Figure 2A).

HT22 cells were labeled in media containing either “heavy” (Lys^8^, Arg^10^) or “light” (Lys^0^, Arg^0^) amino acids for at least six generations. Two sets of heavy-labeled cells were treated with DFO to induce acute and chronic ID. The light-labeled control HT22 cells were incubated with the vehicle (DMSO) for 6 or 24 h, respectively. For the hypoxia treatment, heavy labeled HT22 cells were incubated at 37 °C for 6 h in a hypoxia chamber (1% O_2_, 94% N_2_, 5% CO_2_), while the light-labeled control cells were incubated at 37 °C for 6 h under the normoxia treatment (18% O_2_, 77% N_2_, 5% CO_2_). Proteins isolated from pairs of “light” and “heavy” cells were mixed equally and proteolytically digested with trypsin (Figure 2B). To increase the coverage of phosphoproteomic analysis, peptides were separated using basic pH reverse-phase offline fractionation [40]. Phosphorylated peptides in each fraction were enriched using Immobilized-Metal ion Affinity Chromatography (IMAC, Figure 2C). Peptides with or without enrichment in each fraction were analyzed using Nano-HPLC Orbitrap Fusion mass spectrometer. Biological duplicates were performed to ensure data reproducibility.

### 3.2. Quantitative Analysis of Phosphoproteome in Response to Hypoxia and ID

**Figure 3:**
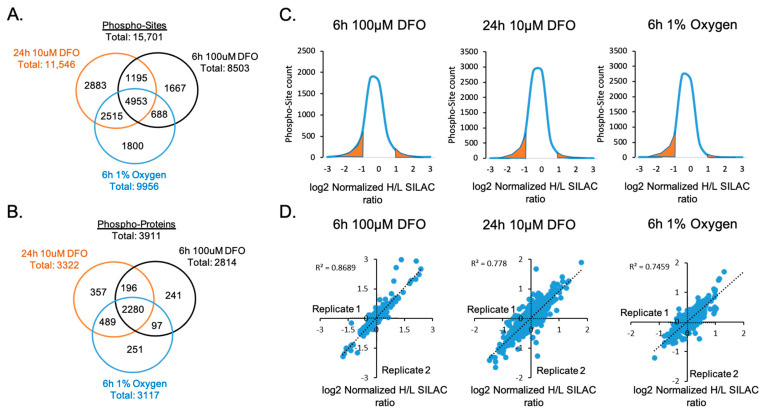
Quantitative analysis of phosphorylated proteins and peptides. Venn diagrams show the number of (**A**) phospho-sites and (**B**) phosphorylated proteins that were commonly or uniquely identified upon 6 h 100 µM DFO, 24 h 10 µM DFO, or 6 h 1% oxygen treatments. (**C**) Distributions of log_2_ normalized phosphorylation site SILAC Heavy/Light (H/L) ratios under each treatment. Highlighted in orange is the area under the curve representing quantifiable phospho-sites with a significant log_2_ normalized H/L SILAC ratio ≤−1 and ≥+1. (**D**) Correlations of replicated quantification of phosphorylation proteome in each treatment.

A total of 15,701 phosphorylation sites on 3911 proteins were identified using FDR <1% (Figure 3A,B and Table S1). Then, 31.5% of the identified phosphor-sites (4953 sites) were shared across all treatments. Under each treatment, more than 10% of quantifiable phospho-sites exhibited at least a two-fold change in relative abundance, indicating that ID and hypoxia significantly affected the phosphorylation signaling pathways in neuronal cells (Figure 3C). Biological replicates showed strong concordance and correlations, demonstrating an excellent reproducibility of the quantification analysis (Figure 3D).

**Figure 4:**
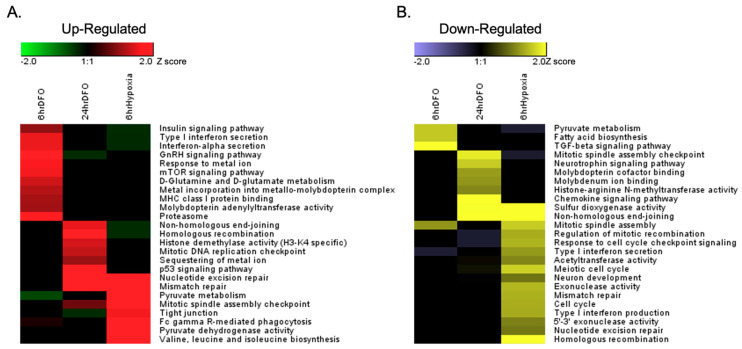
Clustering analysis of the quantitative phospho-proteome datasets with the Kyoto Encyclopedia of Genes and Genomes (KEGG) Pathway. SILAC quantification ratios of all phospho-peptides for each treatment were divided into four quantiles based on the normalized log_2_(Heavy/Light) SILAC ratios (Q1 < −1, −1 ≤ Q2 < 0, 0 ≤ Q3 ≤ 1, Q4 > 1). An enrichment analysis of the KEGG pathway for each quantile was performed using the hypergeometric test with Benjamini–Hochberg adjustment. Phospho-proteome annotations that were significantly upregulated in Q4 (**A**) and downregulated in Q1 (**B**) in each treatment were clustered through hierarchical clustering.

Quantile-based enrichment analysis was performed to cluster overrepresented Gene Ontology (GO) annotations through one-way hierarchical clustering and to compare the differential enrichment of phosphoproteome under each treatment (Figure S1). To account for changes in protein abundance, the phosphoproteome was normalized by the corresponding total protein concentration in each treatment. Our data showed that all three treatments exhibited modifications of similar signaling pathways and unique cellular responses in phosphorylation dynamics (Figure 4).

### 3.3. ID and Hypoxia Induces Global Changes of Phosphorylation Signaling

Acute and chronic ID as well as hypoxia induced both common and disparate effects on protein phosphorylation in neuronal cells, including known hypoxia- and ID-sensitive phosphorylation targets. Both ID and hypoxia lead to increased phosphorylation of HIF1a S652 and BCL2 interacting protein 3 (BNIP3) T86. The phosphorylation of HIF1a S652 by mitogen-activated protein kinase (MAPK) promotes HIF1a nuclear localization and transcriptional activity [41,42,43]. BNIP3 T86 phosphorylation blocks BNIP3-induced cell death upon overexpression by hypoxia and ID [44]. All three treatments resulted in increased phosphorylation of proteins that regulate metal homeostasis. However, these treatments also showed differential changes in phosphorylated proteins, including proteins whose phosphorylation modulates cellular metabolism, inflammatory response, DNA damage and repair, cell cycle control, and epigenetic modifications (Figure 4 and Table 1).

#### 3.3.1. Regulation of Metal Ion Homeostasis

ID resulted in increased phosphorylation of proteins that regulate iron-related cellular processes and enzyme activities. Acute ID was characterized by a significant increase in cellular metal metabolism and protein metalation of the metallo-molybdopterin complex. Similarly, chronic ID increased proteins involved in the intracellular sequestering of iron ions and metal incorporation into the metallo-molybdopterin complex. Both acute and chronic ID lead to a significant increased phosphorylation of Gephyrin, which is a key enzyme in the biosynthesis of the molybdenum cofactor, at S336 and S338. Phosphorylation of both S336 and S338 occurs at known regulatory sites of Gephyrin-dependent neurotransmission [45,46].

#### 3.3.2. Cellular Metabolism

Both ID and hypoxia induced significant changes in the phosphorylation of proteins in the metabolic pathways. Hypoxia increased pyruvate dehydrogenase E1 alpha 1 (PDHA1) phosphorylation at T231 (ratio 9.8), while chronic ID decreased PDHA1 phosphorylation at S300 (ratio 0.5). Both T231 and S300 of PDHA1 are regulatory sites of dehydrogenase activity and mitochondria respiration [47,48]. In addition, 6-phosphofructokinase-L (PFKL) phosphorylation significantly increased under acute ID at S773 (ratio 4.9) and 6-phosphofructokinase-M (PFKM) phosphorylation increased under chronic ID at S667 (ratio 2.1).

#### 3.3.3. Cell Cycle Control

The phosphorylation of proteins mediating cell division processes was significantly decreased under hypoxia but increased under ID. Hypoxia led to the decreased phosphorylation of proteins involved in mitotic spindle assembly and elongation, cell cycle checkpoint signaling, and regulation of mitotic recombination. These include the structural maintenance of chromosomes protein 1A (SMC1A) S358 (ratio 0.4), S360 (ratio 0.5), nucleophosmin (NPM1) S258 (ratio 0.3), and DNA topoisomerase 2-binding protein 1 (TOPB1) S863 (ratio 0.5). An exception was an increase of phosphorylated protein involved in mitotic spindle assembly checkpoint CDK5 regulatory subunit-associated protein (CDK5RAP2) S196 (ratio 1.6). In contrast, ID increased the phosphorylation of proteins involved in mitotic DNA replication checkpoint processes. SMC1A phosphorylation at S358 was increased under acute ID (ratio 9.7), and S360 phosphorylation increased under chronic ID (ratio 8.2). TOPB1 phosphorylation at S863 was also increased under acute and chronic ID (ratios 2.4 and 4.1, respectively).

#### 3.3.4. Regulation of DNA Damage and Repair

DNA repair is a critical cellular pathway maintaining genome integrity. Hypoxia significantly decreased the phosphorylation of proteins in both homologous recombination and non-homologous end-joining pathways, including DNA repair protein RAD50 (RAD50) S236 (ratio 0.4), Replication protein A1 (RPA1) S186 (ratio 0.2) and T189 (ratio 0.2), and exonucleases and endonucleases in DNA repair pathways. In contrast, the phosphorylation of proteins in multiple DNA repair pathways was significantly increased under chronic ID, including mismatch repair, nucleotide excision repair, and homologous recombination. RAD50 phosphorylation at S237 was increased under acute ID (ratio 25.0) and increased at T690 under chronic ID (ratio 6.7). Chronic ID also increased RPA1 S186 phosphorylation (ratio 4.4).

#### 3.3.5. Inflammatory Response

The inflammatory response is intimately linked to iron and oxygen homeostasis and physiology [49,50]. Acute ID increased the phosphorylation levels of extracellular signaling pathways implicated in inflammation. These included type 1 interferon production and secretion high mobility group protein B1 (HMGB1) S100 (ratio 2.5), B2 (HMGB2) S100 (ratio 1.8), and mitochondrial antiviral signaling protein (MAVS) S152 (ratio 2.0). In contrast, hypoxia reduced phosphorylation levels of HMGB1 S100 (ratio 0.3), and B2 (HMGB2) S100 (ratio 0.4), as well as the Toll-like receptor 2 and 4 signaling processes.

#### 3.3.6. Regulation of Epigenetic Processes

ID led to an increased phosphorylation of epigenetic modifiers. Chronic ID increased the phosphorylation of histone H3 lysine demethylase 5C (KDM5C) S301 (ratio 1.7), S317 (ratio 2.2) and 4B (KdDM4B) S972 (ratio 2.1) and decreased the phosphorylation of histone lysine N-methyltransferase ASH1L T726 (ratio 0.5) and SETD2 S890 (ratio 0.5). While they altered histone-modifying proteins to a lesser extent, acute ID and hypoxia uniquely led to significant phosphorylation changes of proteins involved in the regulation of DNA methylation and methyl-CpG binding. Acute ID increased the phosphorylation of cytosine-specific methyltransferase (DNMT1) S140 (ratio 2.9), which is a known alpha serine/threonine-protein kinase (AKT) substrate and a negative regulatory site [51], but it decreased the phosphorylation of methyl CpG binding protein 2 (MeCP2) S216 (ratio 0.6). In contrast, hypoxia increased the phosphorylation of MeCP2 S216 (ratio 1.7), but it decreased the phosphorylation of Ten-Eleven Translocation 2 (TET2) S97 (ratio 0.55), which is an AMPK substrate, reducing TET2 stability and thereby lowering 5 hmC levels [52,53].

### 3.4. Kinase Activity Profiling of Phosphorylation Signaling Dynamics

**Figure 5:**
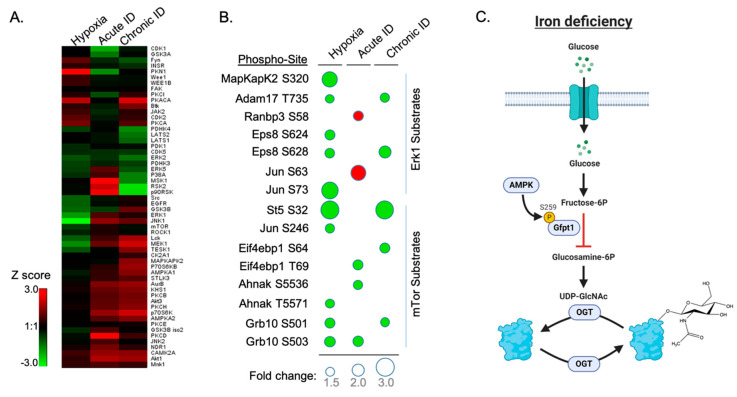
Kinase activity enrichment and clustering analysis of the phospho-proteome dataset. (**A**) Clustering of the Kinase Activity Score of each kinase comparing the kinase activation profile among all three treatments. (**B**) Bubble heat map showing Extracellular Signal-regulated kinase 1 (Erk1) and mTOR phospho-sites were significantly changed under all treatments. Bubble size indicates level of fold change in phosphorylation site with SILAC ratio < 0.66 (Green) and >1.5 (Red). (**C**) Scheme illustrating ID-induced phosphorylation of GFPT1 S259 and thereby reducing the synthesis of glucosamine-6P in the O-GlcNAc pathway.

The objective of this analysis was to identify upstream regulatory kinase pathways whose activities respond to the changes in cellular oxygen and iron availability. Previously published Kinase Substrate Enrichment Analysis (KSEA) with an online portal was limited to the analysis of human kinases and proteomes. To perform kinase activity analysis of our dataset, we developed and utilized a sampling-based statistical approach, termed Kinase Activity Profiling Analysis (KAPA), to evaluate the site-specific changes in the abundance of kinase targets that can be applied to any species with available kinase-substrate databases. For any given kinase, the method applies bootstrap strategy by randomly selecting *k* number of phosphorylation sites (*k* equals the number of target sites of the kinase quantified in the experimental data) and calculating the average SILAC ratio (log_2_ transformed). We included only phosphorylation sites with SILAC ratios that could be normalized by corresponding protein ratios. The sampling process was repeated 1000 times, and the average ratios of each random sampling formed a normal distribution based on the Central Limit Theorem. Then, we can calculate a z-score for the specific kinase based on the average SILAC ratios (log_2_ transformed) of experimentally measured kinase target sites and the normal distribution of averaged SILAC ratios from random sampling. We considered the kinase-specific z-score as the Kinase Activity Score. Positive scores indicated an increase of the phosphorylation level of the identified target sites of the kinase upon the treatment compared to the overall phosphorylation proteome changes under the same treatment, while negative kinase activity scores indicated otherwise. In addition, score absolute values also correlate with the confidence of the measurement, as kinases with more quantified target sites may have a more confident measurement of activities and therefore higher absolute score values. The clustering of the Kinase Activity Score of each kinase allowed us to compare the kinase activation profiles under different treatments. Our data revealed distinct kinase activity profiles under acute and chronic ID as well as hypoxia (Figure 5A).

Chronic ID shared a more similar kinase activation profile with acute ID but still has unique features. Similar to the acute treatment, chronic ID led to the activation of AKT, c-Jun N-terminal protein kinase (JNK1), and AMPK pathways. In addition, chronic ID activated the Protein Kinase A (PKA) pathway while downregulating the Mitogen- and Stress-activated protein kinase 1 MSK1 and Ribosomal S6 kinase (RSK) signaling pathways, including RSK2 and p90RSK. Both MSK1 and RSKs play important roles in the MAPK pathway. In contrast, acute ID activated MSK1 and RSK2 pathways. Acute ID specifically activated specific isotypes in the Protein Kinase C pathways including PKCD, PKCB, and PKCH and downregulated cell-cycle-related CDK1 as well as PKN1, which is a negative regulator of AKT kinase. In contrast to ID, hypoxia activated PKN1 kinase, while it significantly suppressed the JNK1 pathway in neuronal cells. Both hypoxia and chronic ID showed a suppression of mTOR and Erk1 pathways (Figure 5B) but activation of the PKA pathway.

### 3.5. Iron Deficiency Regulates Phosphorylation of the Key Enzyme in Glucosamine Metabolism and Negatively Impacts O-GlcNAc Modification in Neuronal Cells

**Figure 6:**
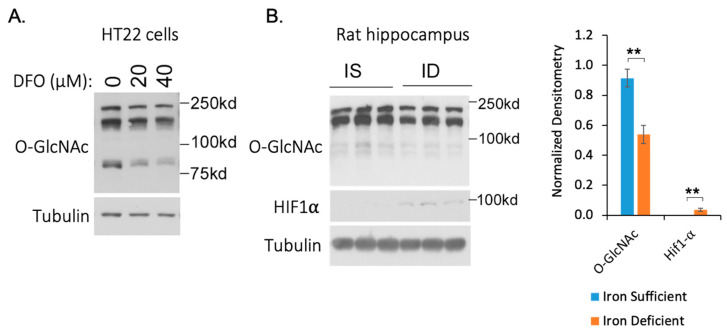
Iron deficiency decreases global O-GlcNAcylation. (**A**) Iron deficiency decreases the global O-GlcNAcylation level in HT22 cells in a dose-dependent manner. HT22 cells were treated with the indicated concentrations of DFO for 24 h and harvested for Western blotting. (**B**) Iron deficiency decreases global O-GlcNAcylation in the rat hippocampus. Iron-sufficient (IS) and iron-deficient (ID) hippocampal protein lysates were analyzed by Western blotting (WB) with the indicated antibodies. Global O-GlcNAc and HIF1a levels were quantified and normalized to tubulin. ** *t*-test, n = 3, *p* value < 0.01.

The predicted increase of AMPK activity led us to evaluate its effect on downstream AMPK targets. AMPK is known to phosphorylate and thereby inhibit glutamine-fructose-6-phosphate transaminase 1 (GFPT1) enzymatic activity [54,55,56]. We observed an increased phosphorylation of GFPT1 S259 under both chronic ID and hypoxia with SILAC H/L ratios of 1.55 and 1.53 respectively, after normalization to the protein abundance. GFPT1 is a rate-limiting enzyme in regulating the flux of glucose entering into the hexosamine pathway through which fructose-6-phosphate (Frc6P) was converted to uridine 5′-diphospho-N-acetyl-d-glucosamine (UDP-GlcNAc), which is a precursor for protein glycosylation substrate glycosaminoglycan (Figure 5C). We hypothesized that the increase in GFPT1 phosphorylation would inhibit the generation of UDP-GlcNAc in neuronal cells and consequently reduce the level of O-GlcNAcylation. To test this hypothesis, we applied DFO to HT22 cells and performed Western blotting (WB) experiments with the anti-O-GlcNAc antibody. Our data showed that global O-GlcNAcylation levels in HT22 cells decreased in response to the DFO in a dose-dependent manner (Figure 6A and Figure S2A). To further confirm this effect in vivo, we performed Western blotting analysis using hippocampi from iron-deficient and iron-sufficient (control) rats. Our data showed that ID decreased O-GlcNAcylation levels in the rat hippocampus accompanied with increased HIF1a levels (Figure 6B and Figure S2B).

## 4. Discussion

Early-life ID results in long-lasting abnormal neurocognitive outcomes in humans [2]. Using animal models, these long-term effects of ID can be explicated in part by profound changes in gene regulation and epigenetic landscape in the brain during periods of robust growth and differentiation [9,57,58]. As such, discovering early response cellular pathways induced by ID is an important step to provide mechanistic insights and therapeutic potentials to mitigate the long-lasting effects in neural development. Cellular ID is known to impair the activity of enzymes that are iron- and oxygen-dependent and increase the activity of specific pathways such as the HIF1a-mediated hypoxia-response pathways to maintain cellular homeostasis [59]. Given that the hypoxia-response pathways are major mechanisms mediated by hypoxic conditions, which occur in neurons due to ischemic brain injury or carbon monoxide poisoning [60,61], little is known at a system-wide level about how these conditions differentially affect the phosphorylation signaling in neuronal cells.

Here, we present the first systematic and quantitative approach to elucidate the changes in phosphorylation dynamics following hypoxia as well as acute and chronic ID in a neuronal cell line. We assessed over 15,000 phosphorylation sites in HT22 cells. Our SILAC-based quantification of biological replicates showed excellent reproducibility and demonstrated high confidence in the reliability of the quantitative analysis, providing a rich resource for the community to study iron and oxygen-dependent phosphorylation signaling in neuronal cells. Importantly, our study revealed rapid and specific changes in global phosphorylation signaling under each condition.

The observed increase in the phosphorylation of proteins involved in metal ion homeostasis is consistent with a previous study showing that DFO treatment decreased the response of genes responsible for dendritic and synaptic development [3]. Gephyrin activity regulates the formation and extension of the GABAergic synapse, thereby affecting the function and availability of neuronal receptors and signaling molecules [62]. Post-translational regulation of gephyrin through CaMKII phosphorylation of S336 and PKA phosphorylation of S338 are known to activate Gephyrin-dependent inhibitory synapse formation [45]. The increased phosphorylation of Gephyrin in response to DFO treatment of HT22 neuronal cells is consistent with the negative impact of ID on hippocampal neuron synaptogenesis and synaptic transmission in vivo [7,31].

Given that iron and oxygen are key substrates for multiple proteins in cellular metabolic pathways [36], hypoxia increased the phosphorylation of proteins involved in pyruvate metabolism, amino acid biosynthesis, and lipid biosynthesis. Conversely, both acute and chronic ID decreased the phosphorylation of proteins in these pathways. Interestingly, the decrease of phosphorylation in pyruvate dehydrogenase (PDH) could lead to increased activity of the PDH complex, indicating an early cellular response by boosting mitochondrial and TCA cycle activity to compensate for a deficit in energy production in iron-deficient cells.

While oxygen and iron regulation share similar metabolic and metal ion homeostasis regulatory control, numerous pathways diverge between hypoxia and ID. In contrast to observed downregulation under hypoxia, ID led to an increased phosphorylation of proteins involved in cell cycle regulation, inflammation, and DNA damage and repair. Hypoxia decreased the phosphorylation of proteins mediating the DNA damage response (DDR) pathways, including both homologous recombinant (HR) and non-homologous end-joining (NHEJ). In contrast, chronic ID increased the phosphorylation of proteins in DDR pathways including HR, nucleotide excision repair (NER) and mismatch repair (MMR), and p53 signaling.

Based on the phosphorylation dynamics under these treatments and existing knowledge on the kinase–substrate relationship, we developed an algorithm (KAPA) to predict the profiles of kinase activity and changes in response to each treatment. The algorithm enables species-independent evaluation of the site-specific phosphorylation dynamics from known kinase substrates. In agreement with our phosphorylation dynamics analysis, KAPA showed distinct changes in kinase activity under each treatment. Acute and chronic ID exhibited more similar profiles compared to the hypoxia treatment and therefore were clustered together. Interestingly, ID led to the activation of AKT, JNK1, and AMPK pathways that are involved in the regulation of energy homeostasis in neuronal cells. These changes further corroborate previous finds of altered mTOR signaling in a neuronal-specific ID mouse model [18]. On the other hand, acute hypoxia showed no change in AMPK and AKT signaling pathways but downregulated JNK1 accompanied by upregulated PKA and PKN1. PKN1 is a negative regulator of AKT signaling. It is likely that acute hypoxia (6 h) was insufficient to perturb the energy balance in cells and therefore did not lead to AMPK activation. However, the causes of such broad and specific differences in kinase activities following each treatment remain to be determined.

ERK1 kinase activity was differentially altered by hypoxia and ID in HT22 neuronal cells (Figure 5A,B). Of note, Adam17 and EPS8 are two substrates of ERK. Adam17 mediates the proteolytic release of several cell-surface proteins, including transforming growth factor-alpha, growth hormone receptor, and the amyloid precursor protein. Phosphorylation at T735 activates Adam17 proteolytic activity [63]. Under oxygen and chronic iron starvation, T735 phosphorylation was downregulated, indicating a decrease in Adam17 proteolytic activity. This decrease in activity has been previously linked to disease progression in Alzheimer’s disease (AD) [64]. The phosphorylation of Eps8 inhibits actin capping activity in actin cytoskeleton remodeling, which is facilitated by BDNF activation of the Erk1 signaling pathways. This activity modulates axonal filopodia formation, which is a process with crucial impacts on neuronal development and synapse formation [65]. S628 phosphorylation was downregulated under oxygen and chronic iron starvation, suggesting abnormal axonal growth. These effects are consistent with previous findings indicating an increased risk of AD and decreased BDNF signaling associated with abnormal axonal growth in early-life iron deficient brains and iron-deficient neurons [3,66,67,68].

O-GlcNAc modification of proteins is an important signaling mechanism that closely links to glucose metabolism in neuronal cells. The dynamics of this post-translational modification is regulated by a single pair of enzymes O-GlcNAc transferase and O-GlcNAcase [69]. O-GlcNAc-modified proteins show an increase in functional activity through the regulation of translocation, DNA binding, transactivation, and protein stability [70,71,72]. O-GlcNAcylation targets are widespread in the brain and are involved in diverse transcription activation and neuronal cell signaling processes [73]. Changes in O-GlcNAc modification have been implicated in cancer, neurodegeneration, insulin resistance, and type 2 diabetes [74,75,76,77]. Our study found that ID led to the activation of AMPK kinase and increased the phosphorylation of its downstream target GFPT1. The increased phosphorylation of GFPT1 led to a reduced pool of UDP-glucosamine, which is the key metabolic precursor for O-GlcNAc modification of proteins, and consequently a lower level of O-GlcNAc modification in iron-deficient HT-22 cells and rat hippocampus. Interestingly, both ferritin and transferrin receptor 1, which regulate cellular iron metabolism, are O-GlcNAcylation substrates [78,79]. It is recognized that our current study of O-GlcNAcylation dynamics was preliminary and limited to Western blotting analysis with RL2 antibody. The broad scope of ID-induced O-GlcNAcylation changes in neuronal cells can be further investigated with the immunoassays of other O-GlcNAcylation antibodies such as CTD110.6 as well as site-specific, quantitative proteomics analysis of O-GlcNAcylation proteome in neuronal cells. While this study presented the first evidence linking cellular ID to O-GlcNAc modification, additional studies are needed to investigate the effects of ID and physiological significance of decreased O-GlcNAcylation modifications on these specific factors and neuronal development. Overall, our study identified diverse cellular pathways differentially regulated in response to these microenvironmental stress and provided a rich resource for the community to study iron and oxygen-dependent phosphorylation signaling in neurons.

## Figures and Tables

**Table 1:** Dynamics of protein phosphorylation pathways regulated by hypoxia, acute, and chronic iron deficiency. Functional activity is summarized either as increased, decreased, or unchanged (blank).

*CELLULAR FUNCTIONS*	HYPOXIA	ACUTE ID	CHRONIC ID
***CELL METABOLISM***	Increase	Decrease	Decrease
***CELL CYCLE CONTROL***	Decrease	Increase	Increase
***DNA DAMAGE AND REPAIR***	Decrease		Increase
***INFLAMMATORY RESPONSE***	Decrease	Increase	Decrease
***METAL ION HOMEOSTASIS***		Increase	Increase
***EPIGENETIC PROCESSES*** *Histone methylation* *DNA methylation*	Increase	Decrease	Decrease

## Data Availability

The data presented in this study are openly available at the ProteomeXchange Consortium open database repository with identifier PXD023294.

## Supplementary Materials

The following are available online at https://www.mdpi.com/2072-6643/13/1/179/s1, Figure S1: Enrichment and clustering analysis of the phospho-proteome datasets based on Gene Ontology and KEGG pathway annotations. Figure S2: Western blotting analysis of O-GlcNAcylation dynamics in response to iron deficiency. Table S1: Quantification of phosphorylation sites with protein normalized SILAC ratios under hypoxia, acute iron deficiency and chronic iron deficiency.
