# Supplementary material for: Iron Deficiency Reprograms Phosphorylation Signaling and Reduces O-GlcNAc Pathways in Neuronal Cells
Source: Nutrients. 2021 Jan 8;13(1):179. doi: 10.3390/nu13010179 (PMC7826960; doi:10.3390/nu13010179)

**Iron Deficiency Reprograms Phosphorylation Signaling and Reduces O-GlcNAc Pathways in Neuronal Cells**

Luke Erber^1^, Ang Luo^1^, Yao Gong^1^, Montana Beeson^2^, Maolin Tu^1^, Phu Tran^2^, Yue Chen^1†^

1. Department of Biochemistry, Molecular Biology and Biophysics, University of Minnesota at Twin Cities, Minneapolis, MN 55455, USA

2. Department of Pediatrics, University of Minnesota at Twin Cities, Minneapolis, MN 55455, USA

^†^Correspondence: Dr. Yue Chen ([YueChen@umn.edu](mailto:YueChen@umn.edu))

**Supplementary Information**

**Supplemental Figure S1.** Enrichment and clustering analysis of the phospho-proteome data sets based on Gene Ontology and KEGG Pathway annotations.

**Supplemental Figure S2.** Western blotting analysis of O-GlcNAcylation dynamics in neuronal cells in response to iron deficiency.

**Supplemental Table S1.** Quantification of phosphorylation sites with protein normalized SILAC ratios under hypoxia, acute iron deficiency and chronic iron deficiency.

**Supplemental Figure S1.** Enrichment and clustering analysis of the phospho-proteome data sets based on Gene Ontology and KEGG Pathway annotations. SILAC quantification ratios of all phospho-peptides were divided into four quantiles based on the normalized Heavy/Light Log2 SILAC ratios (Q1: less than -1, Q2: -1 to 0, Q3: 0 to 1, Q4: more than 1). An enrichment test was performed for (A) KEGG Pathways, (B) Gene Ontology Biological Processes and (C) Gene Ontology Molecular Functions using Hypergeometric Test with Benjamini-Hochberg adjustment. The p values were transformed into z-scores before hierarchical clustering analysis.


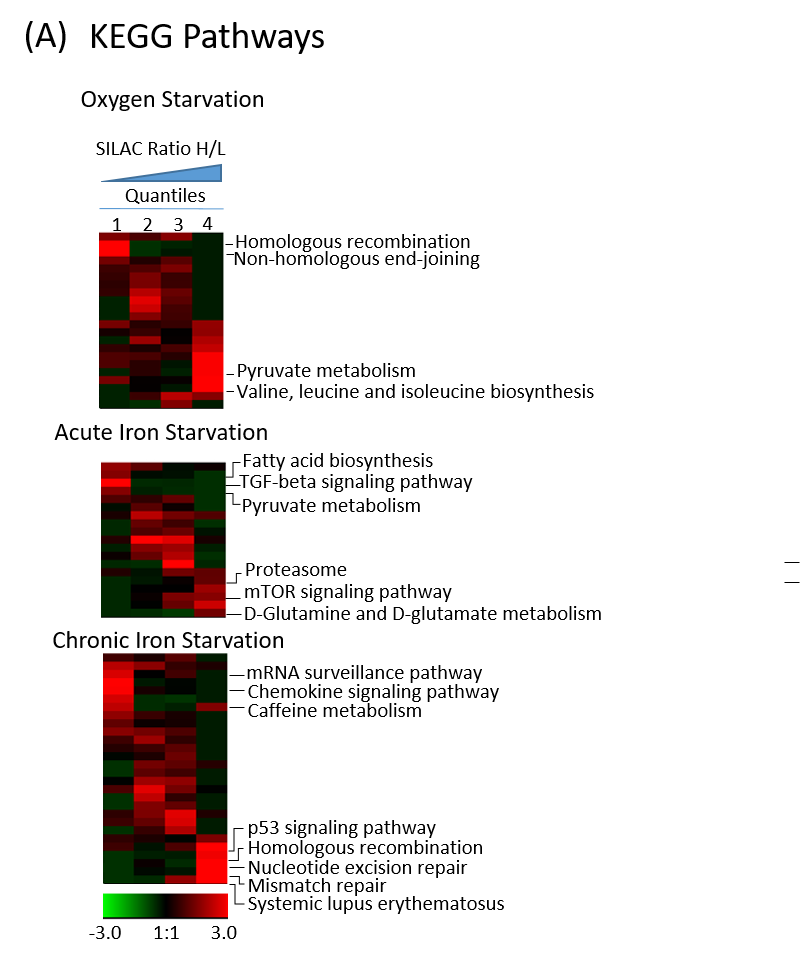


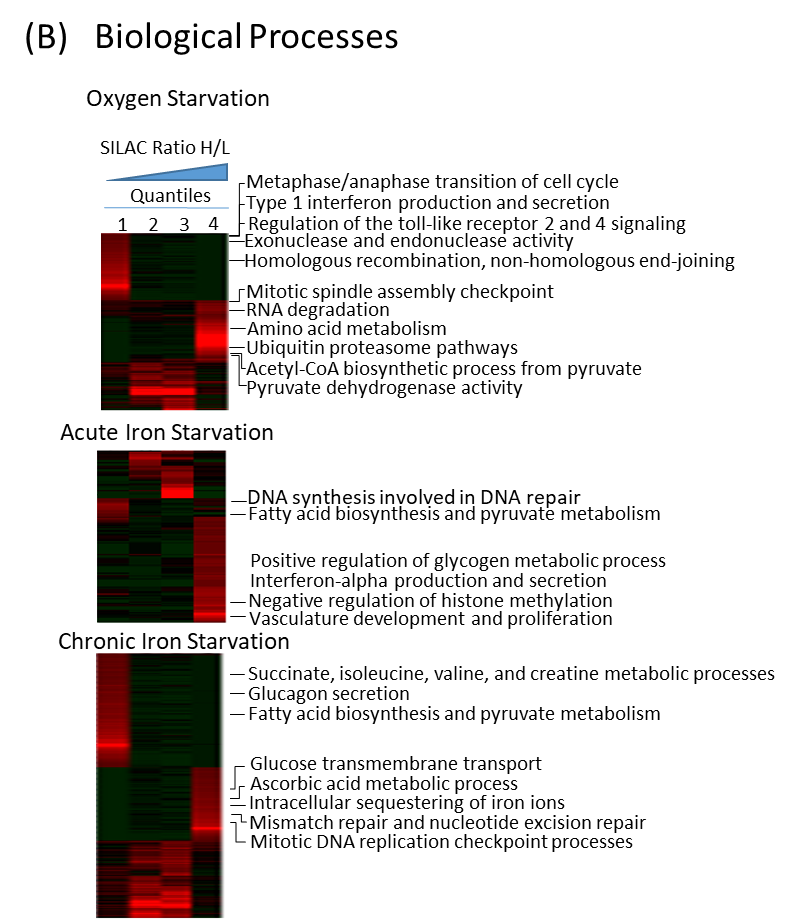


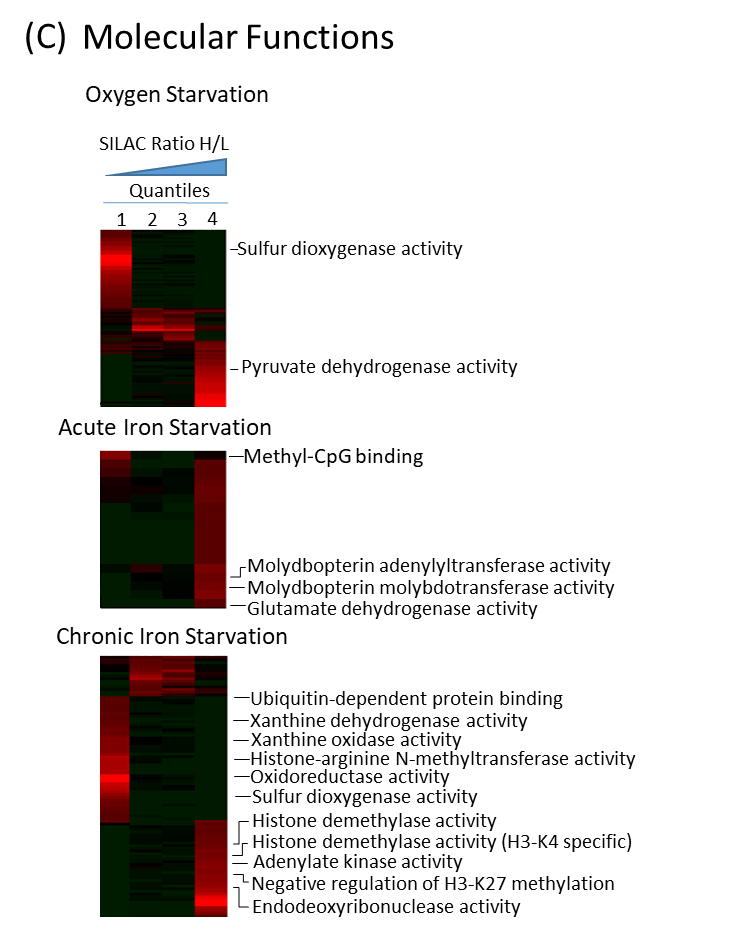


**Supplemental Figure S2.** Western blotting analysis of O-GlcNAcylation dynamics in neuronal cells in response to iron deficiency. Iron deficiency decreases global O-GlcNAcylation level in (A) HT-22 cells and (B) rat hippocampus. HT22 cells were treated with the indicated concentrations of DFO for 24 hrs and harvested for Western blotting. Iron sufficient (IS) and iron deficient (ID) rat hippocampal protein lysates were analyzed by Western blotting.


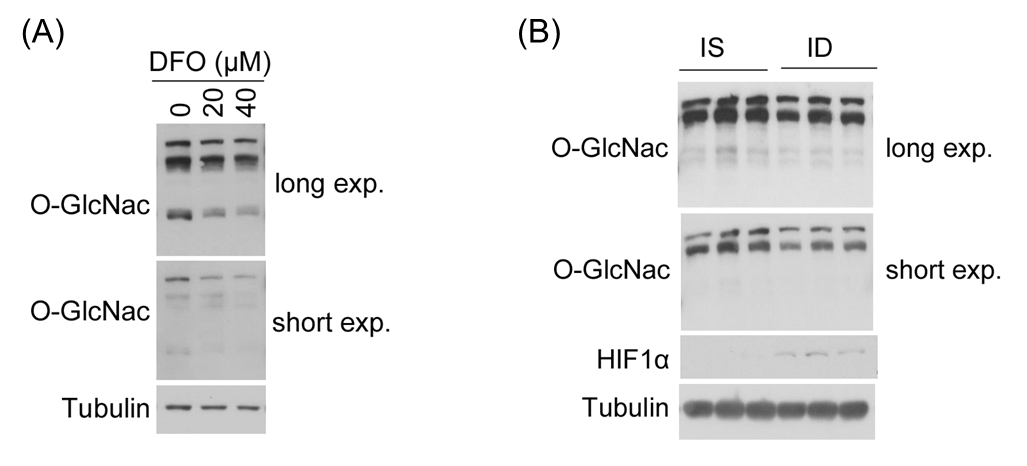

Supplement: Supplementary file 1 [file nutrients-13-00179-s001.zip › Hypoxia phosphoproteomeOnly - combined - Supp-LE - no protein_rev2.docx]
